# Doctors on the move 2: a qualitative study on the social integration of middle eastern physicians following their migration to Germany

**DOI:** 10.1186/s12992-022-00871-z

**Published:** 2022-08-26

**Authors:** Marwa Schumann, Maria Sepke, Harm Peters

**Affiliations:** 1 Dieter Scheffner Center for Medical Education and Educational Research, Dean’s Office of Student Affairs, Charité - Universitätsmedizin Berlin, Free and Humboldt University Berlin, Campus Charité Mitte, Charitéplatz 1, 10117 Berlin, Germany; 2 Medical Education Department, Alexandria Faculty of Medicine, Alexandria University, Alexandria, Egypt

**Keywords:** International migration of health personnel, International medical graduates (IMG), Global health, Middle East and North Africa (MENA), Germany, Social integration

## Abstract

**Background:**

The integration of immigrating physicians has become a challenge for many societies and health care systems worldwide. Facilitating the integration process may benefit both the uptaking country and the immigrating physicians. Previous studies have approached this problem from a system integration perspective. The present study explores the degree of social integration of an exemplary group of Middle Eastern physicians following their migration to Germany from an individual perspective.

**Methods:**

Based on social constructivist epistemology, a series of fifteen interviews and two focus groups with immigrated Middle Eastern physicians (*n* = 23, purposively sampled) were conducted between 2017 and 2020 in Germany. The audio recordings were transcribed, translated into English and analysed deductively based on Esser’s model of social integration, consisting of four dimensions: acculturation, positioning, interaction and identification.

**Results:**

The social integration of the participants showed a multifaceted picture. The early phase was characterized by disorientation and trial and error. Cultural differences were of major importance. Acculturation was facilitated by German language acquisition and increased over time, although some cultural difficulties remained. Professional positioning was facilitated by the need for physicians and a relatively low-hurdle relicensing procedure. Interaction and identification depended on the efforts of the individual physicians.

**Conclusions:**

This study provides a comprehensive picture of the individual social integration of Middle Eastern physicians in Germany. Language and cultural adaptation are identified as being of primary importance. Social integration can be facilitated through orientation programmes or cross-cultural training that benefits the uptaking countries as well as the immigrating physicians.

## Introduction

The ongoing globalization of medicine has had a major impact on health care delivery, workforce training and health policies worldwide [1]. The steadily increasing migration of physicians represents a central feature of this process [1–3]. In parallel to the growing importance of this migration for the affected societies and health care systems, the phenomenon is receiving increasing attention in both politics and the scientific literature [2]. The magnitude and impact of the globalization of medicine are illustrated through the description of this physician migration as “mass migration” that has created a “critical global health workforce crisis” [4, 5]. The direction of this migration generally occurs along the wealth gap, i.e., from less-developed to more-developed countries and from lower-income to higher-income countries [6–8]. The migration of physicians shows some variation in temporal patterns and specific routes in certain regions of the world, e.g., short-term temporary exchange flows versus permanent migration from the Global South to the Global North [9]. Canada, the USA, the UK and Australia represent traditional destination countries with substantial proportions of foreign-trained physicians in their workforces, reaching 23% in the USA, for example [10, 11]. As a result, political economy frameworks that regulate the terms under which foreigners enter the country differ between traditional immigrant receiving countries (e.g., Canada and Australia) and newer countries of immigration (e.g., Germany and Austria), where “legal, administrative, and political mechanisms to control and regulate mass immigration” are still developing [12]. In the following sections, we provide first an overview of the legal and policy frameworks regulating physician mobility at the global, European and German level. This is followed by an illustration of the different theories of integration with a specific emphasis on Esser’s theory of social integration which is used as a framework for this study.

### Legal and policy frameworks

At the global policy level, several international agreements have been put in place to regulate the mobility and recruitment of healthcare workers, e.g., the Global Code of Practice developed by the World Health Organization (WHO), the Health Worker Migration Policy Initiative (HWMPI) and the Global Health Workforce Alliance (GHWA) in 2010 [13]. Following the inception of the European Union, free labour market agreements were issued between its member states, which largely liberated the migration of health care professionals in general and that of physicians in particular [14, 15]. In 1965, the first agreement on intra-European physician mobility was issued, followed by the directive on the free movement of physicians and the recognition of their certificates in 1975. In the same year, the Advisory Committee on Medical Training (ACMT) was established with the responsibility of maintaining high European medical education standards [13].

In Germany, one of the largest member states of the European Union, the uptake of foreign-trained physicians is a relatively new phenomenon, as it is for several other countries in Europe [16]. With regard to the wealth gap and physician migration, Germany is a high-income, industrialized country whose population is constantly ageing [17]. The country has a relatively high density of physicians (1 per 214 inhabitants) [18]. The percentage of migrating physicians increased from 5% in 2006 to 11% in 2016, according to a recent OECD report [19–24]. This represents one of the sharpest increases worldwide.

The health care system in Germany is increasingly characterized by a shortage of physicians, especially in rural areas and in the primary care sector [25]. There is a predicted gap of more than 100,000 physicians in 2030, which equates to approx. 25% of all full-time positions today [26]. Germany has slightly increased its medical school capacity in response to the challenge but not in a manner that would address the physician gap predicted for 2030. Thus, the reliance of the German health care system on foreign-trained physicians is likely to intensify substantially in the future. However, there is still a lack of a systematic international institutional recruitment strategy in Germany [27].

Legal frameworks for recognition in Germany regulated by the federal Ministry of Health (Bundesministerium für Gesundheit) have changed over the last decades; welcoming policies were introduced to facilitate the recognition of professional qualifications and the recruitment of migrant physicians [27, 28]. The Recognition Act for foreign qualifications (Anerkennungsgesetz) was introduced in 2012 with the aim of unifying and simplifying the recognition of foreign qualifications and facilitating access to the German labour market for Third Country migrating physicians [29]. Holding German citizenship is no longer a requirement to obtain a licence to practice medicine; instead, Third Country physicians must pass an equivalence medical knowledge examination in addition to German general and medical language exams corresponding to the B2 level and the C1 level of the Common European Framework of Reference for Languages, respectively [30]. EU doctors, in contrast, obtain an automatic full licence, as their qualifications are automatically recognized within Europe [30].

Therefore, the most common source countries of migrating physicians in Germany are other European countries, e.g., Romania and Greece, followed by Middle Eastern countries [21]. The Middle Eastern region covers southwestern Asia and northeastern Africa, extending from Libya in the west to Afghanistan in the east [31]. The region is characterized by constantly changing political environments, low incomes in many countries and substantial emigration of physicians [32]. For countries such as Germany, the integration of physicians from the Middle East can be perceived as a particular challenge, as the linguistic, cultural and religious differences are substantially larger than those faced in the integration of physicians from other European countries.

As migrant physicians play an important role and will play an even more important role in securing healthcare delivery in Germany in the future, there is a need to facilitate their integration into society and the healthcare system. As this integration process represents a multifactorial process between the specific conditions in Germany and the specific characteristics of migrating Middle Eastern physicians, there is a need for empirical research that reveals what challenges they actually face and what hampers their integration process. Based on a better understanding of those integration problems, society, politicians and organizations can take specific measures to facilitate the integration of foreign-trained physicians [26]. Such measures can improve the placement of Middle Eastern physicians in the healthcare workforce, reduce tension, increase retention rates and increase the quality of patient care [5, 33, 34]. At the individual level, benefits may include higher work satisfaction and improved quality of life for immigrating physicians and their families [4, 35]. Increased self-efficacy and cultural health capital as well as reduced feelings of stress and anxiety have an impact on work, interactions and cultural adjustment [36]. In addition, the internationalization of the medical workforce itself can bring enrichment to the needs of multicultural groups within German society.

### Theoretical background

In the literature and research, there is a debate about the perspective from which the integration processes and experiences of healthcare professionals should be explored best. While some authors prefer the system integration approach that focuses on the integration of the physician workforce on the societal level as a whole, others prefer the social integration approach that examines sociocultural integration on the level of individual actors and their inclusion into the existing social system [18, 37]. From both perspectives, the term “integration” can be defined as “joining parts (in) to an entity,” and its social connotation refers to the “process of mutual accommodation between the majority population and immigrants,” in theory until the point of disappearance of cultural and behavioural differences [38].

To date, much of the literature on the integration of foreign-trained physicians has been written from the system integration perspective, i.e., managing the integration of immigrating physicians into the workforce of a certain health care system at the system level [39]. Several studies have focused on the transition to the workplace, training and job satisfaction of migrating physicians [37]. This perspective emphasizes the so-called “supply side”, for instance, at the level of managing the workforce in a health care system [18, 39]. A prominent example of system integration is the preferential allocation of foreign-trained physicians to rural areas [34].

In contrast, less emphasis has been placed on the social integration processes and experiences of incoming physicians at the individual level, the so-called “demand side” [4, 5, 18, 38], although this perspective seems at least equally important in regard to measuring what facilitates the integration of migrating physicians. In the German context, such research on social integration experience has been undertaken by Klinger and Markmann (2016) on a sample of physicians mainly trained in Romania, Greece and Russia. Given the differences in culture and medical training, their findings may likely be limited in their transferability to physicians trained in Middle Eastern countries [26]. Approaching this topic, a recent study by Los et al. focused on barriers in licence procedures and job application for Syrian-trained physicians following their migration to Germany [25]. While both studies reported in part on social integration experiences, they did not build on an existing integration framework based on theory and empirical studies.

Therefore, this study builds on the theory of social integration by Hartmut Esser, a professor of sociology and philosophy of science at the University of Mannheim, Germany. His work differentiated between system integration, which “refers to the orderly or conflictual relationships between the parts of a social system”, and social integration, which “refers to the orderly or conflictual relationships between the actors” [40]. He developed a theory of social integration that consists of four dimensions of i) acculturation (acquisition of knowledge, language, cultural standards, and competencies); ii) positioning/placement (acquisition and occupation of relevant positions in society and conferral of rights); iii) interaction (establishment of social relations in everyday life, establishment of social networks, cultural capital, and social capital); and iv) identification (emotional attachment to the social system in question via the acquisition of cultural values and orientations) [40]. All four dimensions are interdependent. Placement presupposes a certain acculturation; only with a certain acculturation does placement become possible, and only through this do interaction and identification in a certain social system become possible.

We acknowledge that several other social science theories and frameworks have been developed to explore the integration process of healthcare workers, e.g., Leininger’s theory, including three phases of culture shock, culture stress and cultural imposition, and Pilette’s adjustment theory, including four phases of acquaintance, indignation, conflict resolution and finally integration [37, 41–43]. Sociocultural theories of learning and identity development have also been applied to explore the integration experiences of migrating physicians [44]. Hofstede’s cultural dimensions were developed to explain how cultures differ and include power-distance, individualism–collectivism, masculinity–femininity, uncertainty avoidance, and long-term–short-term orientation [45]. Integration into a host country depends on the similarities/differences of cultures; the smaller the cross-cultural transition is, the smoother the integration [45].

The aim of this qualitative study is to explore the individual social integration of Middle Eastern physicians who have migrated to Germany through the lens of the social sciences. We conducted and analysed a series of interviews and focus groups using a deductive approach based on Esser’s four-dimensional model of social integration as a framework. This article represents a companion study to a recent article that explored the driving forces in a group of Middle Eastern physicians from Egypt preparing to migrate to Germany [46].

## Methods

### Study setting

The study was conducted from June 2017 to June 2020 through face-to-face meetings in various locations in Germany as well as online calls during the COVID-19 pandemic. We conducted fifteen interviews and two focus group discussions with Middle Eastern physicians, each conducted as a single meeting.

For the recruitment of participants, we employed a purposeful, maximum variation sampling strategy to explore a broad range of experiences and maximize the opportunities to elicit data [47]. Recruitment was primarily performed by announcements on social media groups for migrating physicians in Germany. This was followed by a snowballing approach in which participants recommended others who were interested and eligible to participate in this study. Both residents and specialists were recruited. Residents are physicians who were doing their postgraduate training within various specialty fields, and specialists are physicians who had finished their postgraduate training.

### Methodological approach

Our methodological approach was based on a social constructivist epistemology. We chose a qualitative approach, as it would allow an understanding of participants’ perceptions and experiences of their social integration into the German society and health care system in a natural setting with minimal disruption of their daily routines [48]. We conducted an iterative data analysis approach in which data analysis took place concurrently with data collection [49].

The interviews and focus group discussions were audio-recorded. The guiding questions can be found in Appendix. The data were transcribed and translated from Arabic and German into English by the principal researcher (author MSch). ATLAS.ti (a computerized indexing system, GmbH, Berlin, Germany) was employed for transcript analysis.

The translated transcripts were analysed using framework analysis [49]. For the construction of the coding framework, we drew upon the four dimensions of Esser’s model of social integration as a priori items. The principal researcher (MSch) identified the themes and created the initial coding framework based on five interview transcripts, after which data saturation was reached. The senior researcher (author HP) revised the coding framework, and a consensus process followed that involved working with the other researcher (MSch). Finally, coding of the remaining transcripts was continued in the same manner by authors MSch, MSep and HP. Overall, the codes were “revised and amended iteratively to reflect the data” [50].

## Results

### Participants

Fifteen interviews and two focus group discussions were conducted with a total of 23 physicians who were trained in the Middle East. The study participants consisted of 21 residents and 2 specialists from various source countries working in hospitals in different geographic locations in Germany. The small number of specialist participants could be attributed to the fact that Middle Eastern physicians’ migration to Germany has started to increase only since 2011 and reached its peak in 2015 and 2016 [25]. That is why there are still few Arabic speaking specialists in Germany, unlike Turkish physicians, for example, who have been migrating to Germany since the 1960s [29]. The demographic information of the study participants is summarized in Table 1. The length of time the physicians had lived in Germany ranged from 1 to 39 years.

**Table 1:** Demographic data of the participants

Category	Residents	Specialists
Number	*N* = 21	*N* = 2
Gender	Male/Female = 15/6	Male/Female = 1/1
Hospital type	Primary care centres: 1 Secondary tertiary care hospitals: 20	Secondary and tertiary care hospitals: 2
Geographical location	Berlin: 4 Bielefeld: 5 Brandenburg: 2 Herford: 6 Leipzig: 1 Osterode am Harz: 2 Stralsund: 1	Berlin: 1 Herford: 1
Specialty	Anaesthesia: 4 Cardiology: 2 ENT^a^: 2 Gastroenterology and endoscopy: 1 General medicine: 3 General surgery: 1 Internal medicine: 1 Orthopaedic surgery: 1 Paediatrics: 1 Radiology: 2 Neurology: 3	Radiology: 1 Cardiology: 1
Country of origin	Algeria: 1 Egypt: 11 Jordan: 3 Sudan: 1 Syria: 4 Yemen: 1	Palestine: 1 Iran: 1
Age range	28–41 years	54–69 years
Time spent in Germany	1–7 years	7–39 years

^a^Ear, nose, throat

The interviews lasted between 25 and 52 minutes, and the focus group discussions lasted between 42 and 77 minutes.

### Coding framework

The final coding framework was composed of the four dimensions of social integration according to Esser [51]. The themes were acculturation, positioning, interaction and identification (Table 2).

**Table 2:** The four dimensions of social integration according to Esser [27]

Theme	Theme title
1	Acculturation
2	Positioning
3	Interaction
4	Identification

### Theme 1: acculturation

This theme highlights the process of acquiring German language proficiency and knowledge about norms, traditions and customs of the destination country. There was a consensus among the study participants about the importance of the German language as the primary tool to acquire cultural knowledge and become familiar with cultural standards.

*“Speaking the German language fluently makes you able to integrate and adapt; it makes you able to understand how people think. Germans get offended by small things that we see as normal, and vice versa. What we define as rude, they define as normal, so you have to know German culture to be able to adapt.” Female resident, location 1.*

*“It’s the language that allows you to make friends, it’s the language that allows you to fit in with the society, and it’s the language that will enable you to support your family when they join you here. Language is everything. Without the language, there is nothing.” Male resident, location 7.*

The acquisition of cultural competencies occurred through self-learning, trial and error and observation of seniors and peers.

*“I deal with cultural dilemmas through trial and error; I learn that throughout my practice. I also watch how my supervisors deal with such issues and how the nursing staff deals with such cases. I often face cultural dilemmas in intensive care, and that’s when I ask the nursing staff for their help. They know better; they are more experienced. And I also ask my senior colleagues.” Female resident, location 3.*

*“I do a lot of reading about all the things I am experiencing. Why are they celebrating that? What is the background of that holiday season and so on? I educate myself.” Female resident, location 4.*

Most of the participants received no formal cultural preparation, and the participants compared this learning process to being “thrown into cold water”.

*Moderator: “And where did you learn how to deal with different cultures and mentalities?*

*Interviewee 1: “I didn’t learn that. I was thrown into cold water.” Female specialist, location 5.*

While German language proficiency allowed access to cultural knowledge, the physicians still experienced difficulties in some areas. These difficulties occurred in the physicians’ private lives, such as with unfamiliar, country-specific beliefs.

*“I was once in a bakery, and the vendor was cleaning the vitrine. I wasn’t that good at German, so I used sign language instead, and I pointed at the cake I wanted to buy. And she told me not to touch the vitrine. I wondered why she was telling me not to touch the glass. This was rude. In Egypt, it is considered rude if a vendor tells the customer not to touch the glass. But in Germany, it is ok. With time, I came to understand that I should not touch vitrines that had been just cleaned … If you drink coffee in a bakery, you should return the cup, and they will tell you to return the cup … In Egypt we consider this to be rude.” Female resident, location 2.*

*“I remember that once my German colleague was celebrating his birthday within a week, so I simply wished him a happy birthday, but he was very upset. He was in a bad mood the whole day, and he kept shouting at everyone. I didn’t know that Germans believed that early birthday wishes bring bad luck.” Male resident, location 3.*

Such difficulties also occurred regarding traditions, for example, garden work, or the common memory of Germans, such as jokes.

*“I don’t feel integrated because they have a different mentality than ours; they tell a joke, and they burst into laughter, but we don’t even smile. We don’t even understand the joke. First because we come from a different culture and second because we don’t speak that well to be able to understand the joke.” Male resident, location 6.*

Similarly, the physicians lacked knowledge of “old films.”

*“For example, they can talk about old films and laugh about quotes of characters in that film, but I cannot relate to that because I haven’t watched that film. Another example is garden work or taking care of your dog. We are not familiar with these topics or with the tools that they use in their gardens, so when they talk about such topics, I just sit there not being able to participate in the conversation. I simply lack the background about some topics.” Male resident, location 3.*

The physicians also experienced difficulties in the workplace, such as regarding specific beliefs and practices about blood transfusion and vaccination.

*“It was during a shift in the intensive care unit, and I had a patient who refused to take any blood transfusion. I didn’t know what to do. I had no previous experience at all.” Female resident, location 3.*

*“I was confused; I tried to convince the parents about the importance of vaccination, but they were not convinced. In Egypt, I would have called the police. Parents cannot refuse to vaccinate their kids.” Female resident, location 5.*

The physicians also experienced difficulties related to cardiopulmonary resuscitation (CPR).

*“Patients have their rights, and they can refuse some procedures. They can say “No, I don’t want this, I don’t want CPR“, and this is completely new to me. In Algeria, this is impossible for a patient to say “no I don’t want CPR“. This is absolutely impossible. And I still cannot understand that, or accept that.” Female resident, location 8.*

Furthermore, the physicians experienced difficulties associated with minor issues such as using the 24-hour format.

*“I remember once that I made a mistake in documenting the patient’s history. He said that symptoms occurred at 16 o’clock, and I wrote down 4 o’clock because I am not familiar with the 24-hour format. The patient was very upset due to that misunderstanding, and he thought that I didn’t understand what he was saying. He issued a complaint against me.” Female resident, location 3.*

Overall, the participants indicated that the longer they had spent time in Germany, the better their acculturation experiences were.

*“And time; the longer I have lived in Germany, the better I can understand the mentality and culture. They are so different; they have a different lifestyle, different customs and traditions than us. And it takes us time to learn about them. Time and experience make a difference in our integration experience.” Male resident, location 1.*

### Theme 2: positioning

This theme explores the professional positioning and societal positioning of Middle Eastern physicians in the German healthcare system and society, respectively.

Regarding professional positioning, notably, all study participants found employment as physicians in Germany, sometimes even within just a few months.

*“I got a job immediately, after about 1 month, even though I am veiled and I know it’s difficult for a veiled woman to get a job easily. But I got a job only after 1 month.” Female resident, location 8.*

The study participants attributed their successful professional positioning to labour market issues and the increasing need for physicians.

*“I know they invest efforts to integrate me just because they need me. If they didn’t need me, they wouldn’t have recognized my medical certificates. It’s a mutual benefit; what do Germans need from me and what do I need from them. They need me to fill the gap of doctors, and I need the German passport.” Male resident, location 1.*

They also attributed it to an low-hurdle physician relicensing procedure in Germany, which in some cases involves a mere review of credentials.

*“I didn’t have to do any licensing or accreditation exams to be allowed to work as a doctor; I just did a very simple interview. They asked me where I had been working and what my duty was and that was it.” Male specialist, location 4.*

*“I just submitted my curriculum for verification, and I got the permanent licence after one and half years without doing any exams. During this time, I was working with a temporary licence in Bavaria.” Male resident, location 1.*

Despite the smooth process of professional positioning, the Middle Eastern physicians in our sample experienced some challenges when entering the clinical work context in Germany, e.g., an initial loss of professional status.

*“I was originally an ENT specialist in Syria, but now I am working as an ENT resident in Germany.” Male resident, location 6.*

Another challenge the physicians experienced was a need to prove their competence despite having previous experience.

*“When I first came to Germany, I had to prove to them that I was competent enough. Although I had 2.5 years working experience in Egypt, they didn’t trust me until they saw me working.” Male resident, location 1.*

*“It was very difficult at the start; everything was new. I had to learn very quickly, and I dedicated so much of my time to studying. It took me time to gain their trust in me as a physician. They took their time to build up their trust in my knowledge and my skills; they took time to trust me and believe in me. It took them very long, more than a year.” Male resident, location 4.*

*“When I first started working, I was not competent enough, and I was sometimes critiqued.” Male resident, location 2.*

Some problems resulted from professional role confusion and the physicians’ inability to understand the expected behaviour and responsibilities.

*“I didn’t know anything about the rules or the regulations or what was expected from me.” Male resident, location 1.*

*“On my first shift, I had to discharge 4 patients from the hospital. I had absolutely no idea how to write a medical discharge report.” Male resident, location 1.*

*“The biggest example is blood sample withdrawal. This is a minor procedure, and on my first day, they knew I already had some experience in Egypt. But I didn’t know the most trivial procedure of withdrawing a blood sample, because in Egypt it’s not the doctor’s job to withdraw blood samples or insert cannulas, it’s actually the job of the nurse.” Male resident, location 7.*

The physicians also experienced confusion about the organizational features of the German healthcare system, e.g., detailed documentation of the patients’ data.

*“I am not used to writing medical reports … In Germany I was faced with the fact that I have to write so many reports. I had to document every single detail in a computer file, which I had never done in Egypt. I have to explain to the patient every single detail about any procedure, which I didn’t have to do in Egypt.” Female resident, location 4.*

Regarding social positing, the Middle Eastern physicians in our sample valued having equal rights in society and in front of the law as every other German physician.

*“I can remember one particular situation where a patient refused to talk to me because I am a veiled woman. He requested to be examined by another doctor. I felt insulted, but I knew that I was protected by the law. If the patient refuses to be examined by me, then he is obliged to leave the hospital and seek medical treatment in another hospital. Patients have absolutely no rights to choose the physicians they like and reject physicians they don’t like. The law is on my side; it was my shift and my duty.” Female resident, location 3.*

*“And we are all equal in front of the law …*. *I even had to face a medico-legal problem where a patient once sued me in court, but it didn’t matter that I was a foreigner or that I was still a junior resident; it didn’t matter that the patient was German. The case was rejected, and it was very fair.” Male resident, location 1.*

This equality indicated their successful positioning in German society.

Overall, both good social positioning and professional positioning positively impacted the physicians’ integration experience and created a feeling of “fitting in.”

*“I feel I am fitting in when I successfully treat patients and save their lives; I feel I am fitting in when I have achieved professional success. Patients see me as a saviour, and they follow my orders; they do whatever I advise them to do. I am the most important person in the patients’ lives at that moment. In addition, at that moment I feel integrated; I feel that I am the only one who can treat that patient.” Male resident, location 2.*

*“I feel happy when I am competent in doing my job, and that is when I feel that I am fitting in and integrated, for example, when I solve a difficult case of a patient that no one could solve, when I can treat a patient either under the supervision of my senior physician or by myself. I feel a sense of self-fulfilment, and that in turn means integration.” Female resident, location 3.*

### Theme 3: interaction

This theme explores how the study participants interacted in different settings, e.g., in the workplace; in their private or public lives; with different people, such as those from the same or different cultural groups; and in different interaction channels, such as face-to-face or online.

The interactions of the Middle Eastern physicians in our sample showed a large range of experiences, from positive to negative. In interactions with their colleagues at the workplace, the study participants reported experiences of almost mutual acceptance of different eating and drinking habits.

*“And during Ramadan, the head of the department wanted to invite us all for breakfast, but then he recognized that there were 2 of us who were fasting. It was not possible that half of the team would be eating and the other half only watching, so he postponed the invitation. He is understanding. He accepts our choices even if he cannot relate to them and even if he doesn’t understand why.” Male resident, location 9.*

*“We were once celebrating the birthday of a colleague, and they ordered a cake. And they told me we ordered a cake without any alcohol to make sure you can eat with us. They always take my different eating habits into consideration, and I think this is so nice of them.” Male resident, location 2.*

They also reported mutual acceptance of dress codes.

*“And as a woman who wore short skirts and high heels, which was the norm in our department, I was immediately accepted. It was normal to wear short skirts at that time. We all dressed that way. I had no problems at all.” Female specialist, location 5.*

In interactions with patients, the experiences were mixed. On the one hand, patients gave the physicians gifts.

*“A patient once gave me a bottle of wine as a present and then asked me if I was going to drink it. I told him no, so he took it back and brought me another gift; he got me chocolate. The patients are really nice.” Male resident, location 1.*

On the other hand, patients refused to be examined by the Middle-East physicians.

*“I can remember one particular situation when a patient refused to talk to me because I am a veiled woman. He requested to be examined by another doctor. I felt insulted, but I knew that I was protected by the law.” Female resident, location 3.*

Interactions in private life also showed a large spectrum; some study participants were more open about and successful in building relationships with German neighbours and friends.

*“We (the participant and her neighbours) have a very good relationship, especially with my kids. My kids and their kids go to the same school, so we have a good relationship.” Female resident, location 8.*

*Moderator: “Did you make new friends in Germany, or are these your old friends from college?”*

*Interviewee 2: “Most of them are old friends from college days who also moved to Germany, but I also made some new friendships with other Egyptians living here.” Male resident, location 2.*

*“When I first came to Germany, I had only one friend that I had known before, so I started building my network through his network of friends and contacts. He used to introduce me to his friends, German as well as Egyptian friends. I was trying to integrate as much as I could, trying to build my network of contacts. I took each and every chance to go out with people; I used to ask him, “Can I join please?” And he let me join him in his outings with friends. I joined them in all the different places, in bars, in restaurants; there are so many things that I had never done before but did for the first time in Germany. So, I made many contacts in a very short time. I feel well integrated because I have put so much effort into integrating. I go out every single day. I am really grateful for my friend who introduced me to all his friends.” Female resident, location 4.*

Some participants even married German spouses.

*“The fact that I was married to a German; I was familiar with the German way of living even at home. And when I compare myself to my relatives who have also migrated to Germany but live only among Persian circles, they definitely have more difficulties than I do. Inside, they were still living in Iran, but me, I wasn’t living in Iran anymore. On the inside, I was living in Germany because I was married to a German.” Female specialist, location 4.*

Others reported preferring to interact almost exclusively with people from the same national/ethnic group.

*“When I return home from work, I return to the Arab world; I speak Arabic with my family, and I don’t need to interact with German society anymore. And we live among Arab circles only; when we were in Berlin we communicated in Arabic only. We registered in an Arabic-speaking driving school, and we even took the driving test in Arabic.” Male resident, location 6.*

Online interactions played a minor role compared to face-to-face interactions. Some study participants used two secret and separate Facebook groups, with one for females and one for males, where they discussed their problems and challenges.

*“After we migrated, we only had 2 secret Facebook groups, one for the female doctors and the other for the male doctors. We discuss sensitive topics and sometimes consult each other about patients’ confidential issues, and we might face legal action if the groups were not secret.” Male resident, location 5.*

Overall, smooth interactions were mainly facilitated by personal efforts to build relationships.

*“I did build a network of relations. I even connected with people who hardly spoke to each other. I organized outings with my German colleagues who had never gone out together in their lives. I created a network of friends and not only colleagues.” Male resident, location 3.*

However, efforts to build relationships did not equate to successful acculturation for all immigrating physicians. Some participants reported difficulties regardless of the amount of time spent in Germany, such as avoidance of contact and isolation rather than engagement in open communication about cultural dilemmas.

*“And people on the street think we are refugees because we have dark skin like refugees. They think we are refugees, so we will still be considered foreigners even if we have a German passport.” Male resident, location 2.*

*“And they have totally different customs and traditions, which makes it very difficult to find common interests. Their leisure activities are different, their celebrations are different, they drink alcohol, and this is not allowed in our traditions. That’s why it is difficult to find common ground. So, I distance myself to avoid invitations for food or for dinner because then I have to refuse.” Male resident, location 3.*

Interestingly, this study was conducted at a time when the interactions of Middle Eastern physicians in all areas were affected by the large influx of refugees into Germany in 2015. The study participants described facing prejudice as a result of being mistaken as refugees. The prejudice was strengthened by news in the media and resulted in many Germans’ primary perceptions of all Arabic-looking people as refugees.

*“There is a huge amount of propaganda about negative examples of foreigners or Arabs. And this affects the way people look at me and treat me. When people see me on the street, they immediately recognize that I am a foreigner, and they put me in that stereotype; being a foreigner means I am not well educated. People believe the negative image of foreigners in the media, and this is a big barrier against our integration.” Male resident, location 1.*

*“Germans get shocked when they see people who have a different look, but they only get shocked for a few seconds, and they start acting normally thereafter. The problem of the refugees has complicated our situation; we are considered refugees until proven otherwise.” Male resident, location 1.*

### Theme 4: identification

This theme explores the participants’ development of identification with German values, traditions and culture, which for some extended to a feeling of being a German. The study participants reported a wide range of perceptions regarding their identification. Complete belonging and identification with German culture was perceived both by specialists who had already spent more than 20 years in Germany and by those who had acquired German citizenship or who were married to a German spouse.

*“I feel well integrated in Germany because I have a job here and my children go to German schools. And I also have a German passport, so I can say that I feel well integrated … I have lived long enough in this country to become familiar with its culture.” Male specialist, location 3.*

*“The fact that I was married to a German. I was familiar with the German way of living even at home. And when I compare myself to my relatives who have also migrated to Germany but live only among Persian circles, they definitely have more difficulties than I do. Inside, they were still living in Iran, but me, I wasn’t living in Iran anymore. On the inside, I was living in Germany because I was married to a German.” Female specialist, location 4.*

Others felt that they would remain foreigners despite their acquisition of German citizenship.

*“Being part of German society will never happen. We will always remain foreigners who live in Germany and respect the German law, but we will never become German citizens.” Male resident, location 1.*

*“My name is not Schneider or Meier; I am not blond, and I don’t have green eyes. I will never become a German citizen. Even the children who were born and brought up here are not considered Germans. It’s not a matter of passport; it’s a matter of race. And we will never belong to the German race.” Male resident, location 2.*

*“And people on the street think we are refugees because we have dark skin like refugees. They think we are refugees, so we will still be considered foreigners even if we have a German passport.” Male resident, location 2.*

These participants attributed this permanent foreigner status to a lack of “common ground,” cultural values and belonging.

*“And they have totally different customs and traditions, which makes it very difficult to find common interests. Their leisure activities are different, their celebrations are different, they drink alcohol, and this is not allowed in our traditions. That’s why it is difficult to find common ground. So, I distance myself to avoid invitations for food or for dinner because then I have to refuse.” Male resident, location 3.*

*“I went to a Christmas celebration only once in my life. And I felt I didn’t belong there; I felt like a stranger. I didn’t like the way they were celebrating or the way they were drinking or dancing; I didn’t belong there. This is not my place. This was my first and last time to attend a Christmas celebration at work.” Male resident, location 3.*

Identification with the German culture was sometimes perceived in a negative way among the same national/ethnic group.

*“Syrian girls here (in Germany) are very strange; they refuse to get married, and they want to finish their education first. They have become more independent. And that’s why I cannot find a suitable girl, because their mentality has changed.” Male resident, location 6.*

## Discussion

Along with the globalization of medicine and healthcare, both the system and social integration of foreign-trained physicians have become a growing challenge that many countries are facing at the turn of the twenty-first century. To inform better management of this challenge, the present study explored and shed light on the social integration processes and experiences of a sample of Middle Eastern physicians following their migration to Germany. Using Esser’s theoretical framework of the four dimensions of social integration allowed us to form a comprehensive and detailed picture of the individual social integration processes and experiences of these physicians. In the following section, we will discuss our findings according to the social integration subdomains of acculturation, positioning, interaction and identification and in light of the literature. In addition, we will derive recommendations that could facilitate social integration based on the problems identified in our sample of migrating Middle Eastern physicians.

Regarding the acculturation dimension of social integration, the quotes of the study participants indicate that the acquisition of language proficiency and adaptation to cultural norms and customs improved with increased time spent in Germany. The acquisition of the German language was perceived as the most important tool to acquire cultural knowledge and become familiar with cultural standards. This finding is consistent with those of previous studies that showed that insufficient language skills and cultural competencies affected the perceived professional competence of physicians, creating a sense of insecurity and doubts among patients as well as colleagues [39, 52–54]. In addition to the reported difficulties in building relationships, inadequate language skills may also cause unnecessary risks in the work of physicians, including misdiagnosis or treatment [20]. Our participants reported acquiring their cultural competencies through self-learning, trial and error and observation of seniors and peers. They had received no formal cultural preparation. Thus, one recommendation of this study is that emphasis should be placed on both language and cultural training. Such training may enable foreign-trained physicians to work in cross-cultural environments and ensure safe medical practice [39, 55].

Regarding the positioning dimension of social integration, the participants generally felt well positioned in their workplaces and in their work as physicians. Their good workplace integration was mainly attributed to the increased need for physicians in Germany and the relatively low-hurdle relicensing procedures. This finding is comparable to the results of previous studies [27, 46]. Compared to the very difficult relicensing processes in other countries, Germany has a less difficult but often slower and more bureaucratic process [25, 26]. After proving the necessary German language skills, non-European physicians either apply for an equivalency assessment of their credentials by a medical expert or take a practical medical exam to obtain a permanent medical licence [25]. In the USA, for example, international medical graduates have to pass the 3 steps of the USMLE and pay approximately 4000 US dollars before being allowed to practice medicine [56]. Germany’s relatively low-hurdle and inexpensive relicensing procedure may be related to the fact that the immigration phenomenon is relatively new and is taking place on a rather small scale in Germany. In contrast, foreign-trained physicians can face longer periods of unemployment in countries such as Canada and the UK or can face deskilling, devaluing and even an inability to work as a healthcare professional in countries such as Belgium and Austria, where relicensing is difficult and substantial proportions of immigrating physicians contribute to the workforce [57–59]. The easy and inexpensive relicensing procedure can be considered an advantage of Germany as a destination country attracting migrating physicians. In turn, this relicensing procedure may in the future lead to concerns regarding its effects on the quality and safety of the German healthcare system.

Regarding their professional positioning, the Middle Eastern physicians in our cohort also reported difficulties such as their initial disorientation, loss of professional status and professional devaluation, in line with previous studies [33, 39]. Thus, another recommendation of this study is that emphasis should be placed on the incorporation of social support measures for migrating physicians, for instance, in the form of mentoring relationships and buddy systems for peer support, especially among junior-level trainees [60].

Regarding the interaction dimension of social integration, we observed a heterogeneous picture as well. In the workplace, the study participants reported predominantly positive experiences in their interactions with colleagues and patients, although rejection based on intercultural aspects was experienced as well. These findings are in line with previous research on immigrating physicians in Canada and the USA [36, 60, 61]. Outside the workplace, some Middle Eastern physicians also succeeded in building relations with Germans, which was mainly based on the individual efforts of the physicians. On the other hand, some study participants preferred to interact within social networks of the same national/ethnic group, which created an imbalance between the cultures of the host country and the country of origin, leading to segmentation rather than full integration [51].

Interaction only within one’s national/ethnic group may also create a sense of “cultural separateness” and “otherness” and feeling “like an outsider,” leading to social isolation, which hinders full integration into the destination country [5]. Some negative interaction experiences included discrimination by patients. This finding is in accordance with previous studies from Germany, Australia, the USA and the UK [26, 34, 39, 58, 62]. In our sample, however, the experience of discrimination was limited to some patient encounters and did not include colleagues, nurses or everyday life situations. In contrast to the well-established role of social media in the premigration phase, our participants valued real-life interactions more than virtual communities [46].

In line with previous research, these findings lead to the recommendation to emphasize the crucial role of migrating physicians’ interactions with host country nationals and adjustment to the culture of the host country, especially in the initial transition phase. These goals can best be achieved by improving the organizational intercultural awareness of doctors, nurses and patients and ideally by enhancing intercultural competencies through training programmes with immigrants and nationals [36].

Regarding the identification dimension of social integration, the participants’ emotional attachment to the German social system and values involved a broad spectrum of experiences, ranging from a complete sense of belonging (e.g., after acquiring German citizenship or marrying a German spouse) to a feeling of not fitting in and being a complete stranger due to the wide cultural gap between the Middle East and Europe. This finding may be related to the aforementioned deficiencies in acculturation and interaction achieved. Thus, the recommendation for this dimension of social integration is to improve the activities that facilitate the acculturation, positioning and interaction of the migrating physicians. More socially involved migrants are more likely to find friends or partners, improve their language more easily and acquire cultural behaviour more quickly.

Esser’s model of social integration was chosen as a social science framework to explore and analyse the multifaceted integration process in our group of Middle Eastern physicians in Germany. In line with Esser’s framework, the findings of our study underscore that the four dimensions are interrelated; for instance, acquiring language proficiency is a prerequisite to acquiring knowledge about the norms, traditions and culture in Germany. Some degree of acculturation is required to facilitate placement in the workplace and in society as well to establish and develop relationships with German citizens. Success in acculturation, placement and interaction enables Middle Eastern physicians’ identification with Germany. Our study extends the work of previous studies focusing on the system-based integration of foreign-trained physicians into the workforce of a country’s health care system [40]. In addition, Esser’s framework of social integration allows a more comprehensive, detailed and actionable analysis of the integration process and experiences of migrating Middle Eastern physicians than the frameworks introduced by Leininger, Pelitte and Hofstede.

One limitation of this study concerns the language; the interviews and focus group discussions were conducted in different Arabic and German dialects according to the participants’ preferred languages and were translated to English for analysis purposes. The translation process may have altered the original meanings, which were constructed through the physicians’ native language rather than expressed via translation [63]. Translation was also a barrier to a full discourse analysis, a step that would have ensured a more accurate linguistic interpretation of the data [54]. A further limitation includes possible selection bias; our study sample was mainly composed of physicians from Middle Eastern countries, which are the source countries strongly represented by migrant physicians in Germany [21]. Physicians from other source countries have been included in other studies [39].

## Conclusions

Empirical research can contribute to a better and more actionable understanding of the globalization of medicine and the migration of physicians. This study provides a comprehensive and detailed picture of the individual social integration processes of Middle Eastern physicians following their migration to Germany. The study introduced the social integration theory and framework to a larger international audience and researcher group. In terms of all dimensions of the Esser framework, physicians’ degrees of social integration can be characterized as heterogeneous. The key facilitators of the social integration of Middle Eastern physicians include German language acquisition and the increased need for physicians and the relatively low-hurdle relicensing procedure in Germany, the host country.

As migrant Middle Eastern physicians will play an increasingly important role in securing healthcare delivery in Germany, this study provides a better understanding of their specific integration problems. Based on its results, German society, politicians and organizations can take specific measures to facilitate the integration of this group of highly skilled healthcare workers. Areas where the social integration process could be improved include physicians’ initial professional disorientation and role confusion as well as their social isolation. Creating a culturally rich working environment should be achieved at the organizational, training and individual levels, preferably prior to physicians’ starting work, at the beginning of their practice, and throughout their employment [36].

Approaches to creating such an environment can be realized on the macro, meso and micro levels, which involve policy makers, hospital management and individual care teams, respectively [36, 39]. Labour market access policies, mentoring schemes, organizational induction, peer support groups, buddy programmes, supervised practice, “observerships” and ongoing evaluation of migrating physicians’ adjustment are concrete examples of achieving integration on the three aforementioned levels [36, 39]. The coexistence of staff of different educational, professional and cultural backgrounds in the healthcare system may be challenging to manage; however, such diversity is considered a strategic asset and an enriching aspect that could improve the quality of the health care services delivered [18].

## Data Availability

The datasets used and/or analysed during the current study are available from the corresponding author upon reasonable request.

## Appendix

### Discussion guide

- How did you get the German Approbation?

- Did you do a medical knowledge exam/the German medical language exam? What is your opinion about it?

- Have you changed your position before? If yes, can you tell us more about it?

- After living in Germany and working in the German health care system, are your expectations confirmed or contradicted, and why?

- How do you feel as a foreign medical doctor in the hospital? To what extent do you feel integrated/not integrated with the team?

- What has facilitated/inhibited your integration?

- Can you tell one story about a time when you felt truly integrated/not integrated with the team you are working on, the hospital you are working at or the community in general?

- What is your biggest challenge/the biggest benefit you are experiencing?

- Do you recommend that your colleagues work in Germany? If yes, what would you recommend that your colleagues do in order to prepare themselves to work in Germany?
